# Genomic Insights into Marburg Virus Strains from 2023 and 2025 Outbreaks in Kagera, Tanzania

**DOI:** 10.3201/eid3201.251314

**Published:** 2026-01

**Authors:** Lawrence A. Mapunda, Medard Beyanga, Nyambura Moremi, Jean N. Hakizimana, Doreen Kamori, Alfred Chacha, Edna Mgimba, Dennis Mrosso, Ambele E. Mwafulango, Jackson Mushumbusi, Ferdinand Ndunguru, Seif Abdul, Emmanuel Mkumbo, Maria E. Kelly, Céline Barnadas, Vida Mmbaga, Rogath Kishimba, Samwel Laizer, Ntuli Kapologwe, Michael Kiremeji, Erasto Sylvanus, Angela Samwel, Saumu Nungu, Emmanuel Achol, Julien Nguinkal, Hakimu Idris Lagu, Muna Affara, Florian Gehre, Calvin Sindato, Chacha Mangu, Elias Nyanda Ntinginya, Saida Murugwa, Pawan Angra, Shannon Whitmer, George Mgomella, Mahesh Swaminathan, Wangeci Gatei, Dorcas Wanjohi, Sofonias Tessema, Yenew Kebede, Said Aboud, Charles Sagoe-Moses, Alex Magesa, Gerald Misinzo, Tumaini Nagu, Grace Magembe

**Affiliations:** National Public Health Laboratory, Dar es Salaam, Tanzania (L.A. Mapunda, N. Moremi, A. Chacha, E. Mgimba, D. Mrosso, A.E. Mwafulango, J. Mushumbusi, F. Ndunguru, S. Abdul); Ministry of Health, Dodoma, Tanzania (M. Beyanga, E. Mkumbo, V. Mmbaga, R. Kishimba, S. Laizer, N. Kapologwe, M. Kiremeji, E. Sylvanus, A. Samwel, S. Nungu, A. Magesa, T. Nagu, G. Magembe); Sokoine University of Agriculture, Morogoro, Tanzania (J.N. Hakizimana, G. Misinzo); Muhimbili University of Health and Allied Sciences, Dar es Salaam (D. Kamori); World Health Organization, Dar es Salaam (M.E. Kelly, C. Sagoe-Moses); World Health Organization, Geneva, Switzerland (C. Barnadas); East African Community, Arusha, Tanzania (E. Achol, J. Nguinkal, H.I. Lagu, M. Affara, F. Gehre); Bernhard Notch Institute for Tropical Medicine, Hamburg, Germany (M. Affara, F. Gehre); National Institute for Medical Research, Dodoma (C. Sindato, C. Mangu, E.N. Ntinginya, S. Aboud); US Centers for Disease Control and Prevention, Dar es Salaam (S. Murugwa, P. Angra, S. Whitmer, G. Mgomella, M. Swaminathan, W. Gatei); Africa Centres for Disease Control and Prevention, Addis Ababa, Ethiopia (D. Wanjohi, S. Tessema, Y. Kebede)

**Keywords:** Marburg virus, Marburg virus disease, viruses, outbreaks, zoonoses, Filoviridae, genomes, viral hemorrhagic fever, Kagera, Tanzania

## Abstract

Marburg virus (MARV) is the primary cause of Marburg virus disease (MVD), a severe hemorrhagic fever with a high case-fatality rate. The first reported MVD outbreak in Tanzania occurred in 2023, followed by a second outbreak in 2025, both within the Kagera region. During those MVD outbreaks, 174 suspected cases were identified; of those, 10 were laboratory confirmed. After complete genome assembly and bioinformatic analyses, we found the MARV strains of the 2023 and 2025 outbreaks to be closely related and clustered with MARV strains that caused outbreaks in Rwanda (2024) and Uganda (2014). The sequences from both MVD outbreaks in Tanzania showed >99.71% nucleotide identity, suggesting a possible single spillover event followed by limited human-to-human virus transmission. Further ecologic studies are essential to identify potential spillover events, but our findings indicate that closely related MARV strains circulate in Kagera, Tanzania, posing a risk for future outbreak recurrence.

Viral hemorrhagic fevers (VHFs) are zoonotic or vectorborne diseases that pose a substantial threat to global health security and cause substantial economic burdens because of their rapid transmissibility and high fatality rates, as well as the limited availability of effective therapeutics and countermeasures. The incidence of VHFs is notably high in the tropical regions because environmental conditions favor the presence of vectors and reservoirs, as well as increased interactions between humans and animals (*1*). In addition, the threat of spread is magnified by delayed confirmatory diagnosis of VHFs in resource-limited settings, human displacement resulting from conflicts, and rapid increase in intercontinental travel and trade (*2*).

Marburg virus disease (MVD) is one of the VHFs caused by 2 orthomarburgviruses, Marburg virus (MARV) and Ravn virus (RAVV), classified into a single species, *Orthomarburgvirus marburgense*, genus *Orthomarburgvirus* of the *Filoviridae* family (*3*,*4*). MARV was the first filovirus discovered in 1967 during a concurrent outbreak of hemorrhagic fever among laboratory workers in Yugoslavia (Serbia) and Germany who had been in contact with tissues from African green monkeys imported from Uganda (*5*,*6*). The genome of MARV is composed of a nonsegmented negative-sense RNA with ≈19 kbp nucleotides. Genome sequencing is useful in MVD surveillance, contact tracing, and tracking the evolution of MARV and RAVV (*7*).

The Egyptian rousette bat (*Rousettus aegyptiacus*), a cave-dwelling bat, is the primary natural reservoir of MARV; transmission to humans occurs through spillover events from infected bats or contact with their bodily fluids or tissues (*8*). Outbreaks of MVD have frequently been associated with visits to bats’ roosting sites, such as caves and mines, particularly within the geographic range of *R. aegyptiacus* bats (*9*,*10*). Two notable MVD outbreaks were reported in Africa: the 1998–2000 outbreaks in the Democratic Republic of the Congo (DRC), which resulted in 154 cases and 128 deaths (83% case-fatality rate [CFR]), and the 2004–2005 outbreak in Uige, Angola, which resulted in 252 cases and 227 deaths (90% CFR) (*10*–*13*). More recently, the third largest outbreak of MVD was reported in Rwanda in 2024, causing 66 confirmed cases and 15 deaths (23% CFR) (*14*). Other isolated MVD outbreaks have been identified in South Africa (1975), Kenya (1980, 1987), Russia (1990), Uganda (2007, 2008, 2012, 2014, 2017), Guinea (2021), Ghana (2022), and Equatorial Guinea (2023) (*15*).

The first MVD outbreak in Tanzania was declared in Kagera region in March 2023 and consisted of 9 cases (8 confirmed and 1 probable) and 6 deaths (66.6% CFR) (*16*). A second outbreak occurred in the same region in January 2025 and consisted of 10 cases (2 confirmed and 8 probable) and 10 deaths (100% CFR). In this article, we present genomic analyses and examine the genetic relationships among the MARV strains responsible for the 2023 and 2025 outbreaks in Tanzania and previously reported MARV strains reported in both humans and animal reservoirs. The Medical Research Coordinating Committee of the Tanzania National Institute for Medical Research granted ethics approval (certificate no. NIMR/HQ/R.8a/Vol.IX/4957) and permission to publish (ref. no. BD.242/437/01C/42).

## Methods

### Study Settings

**Figure 1:**
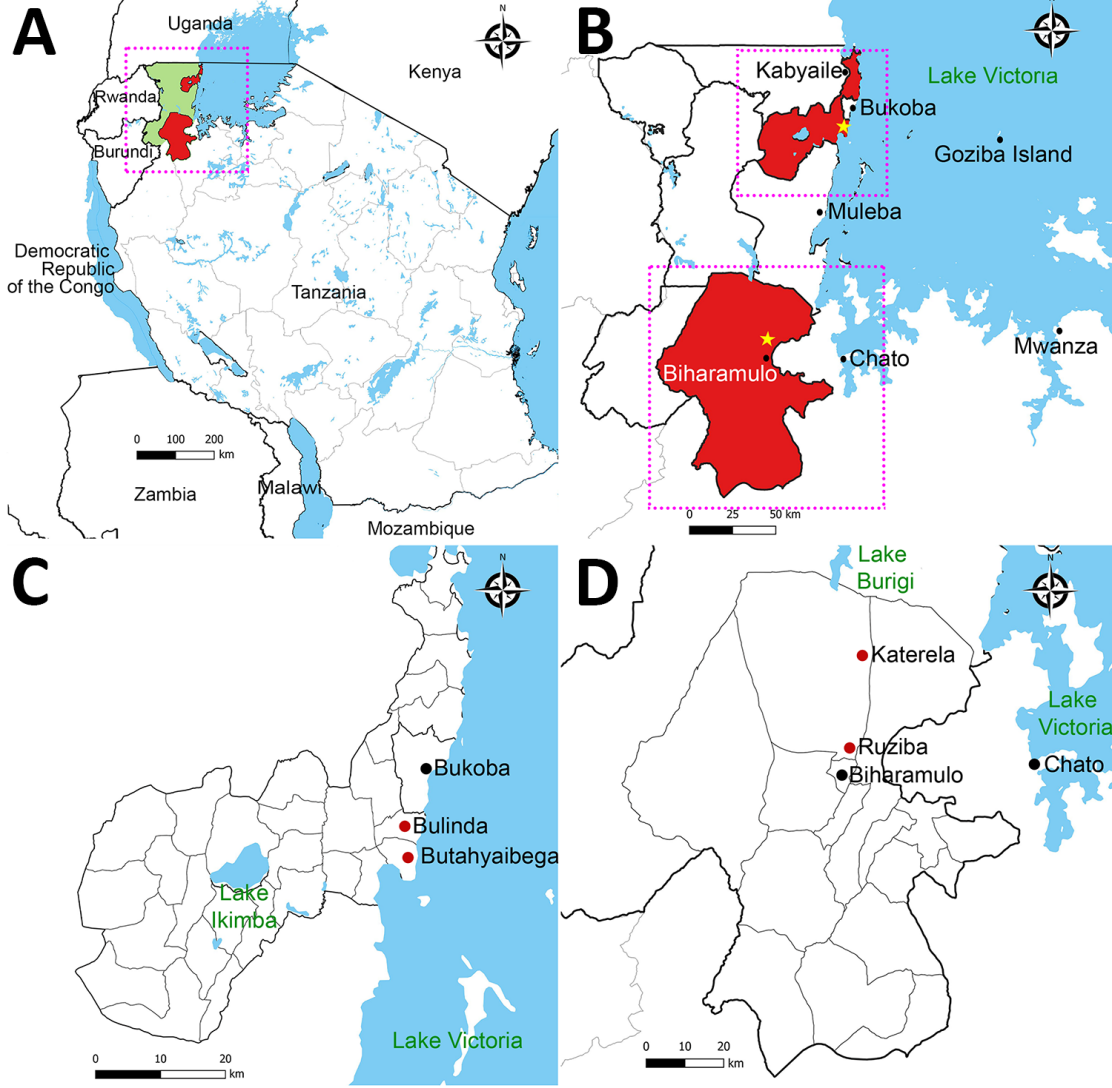
Locations of the 2023 and 2025 Marburg virus outbreaks in Kagera region, northwest Tanzania. A) The outbreaks occurred in northwest Tanzania, west of Lake Victoria, in Bukoba Rural and Biharamulo districts of the Kagera region. The region borders Uganda, Burundi and Rwanda. Green shading indicates Kagera region. Red shading indicates the affected districts. B) Expanded view of boxed area from panel A shows epicenters for both outbreaks (stars). Upper box marks the location of the 2023 outbreak in a village south of Bukoba town; lower box marks the location of the second outbreak in a village north of Biharamulo town. C) Locations of the 2023 outbreak in Bukoba Rural district. Red dots indicate affected villages. D) Locations of the 2025 outbreak in Biharamulo district. Red dots indicate affected villages.

The 2023 and 2025 outbreaks of MVD in Tanzania occurred in the Kagera region, northwestern Tanzania. The index case of the 2023 MVD outbreak was in a resident of Butahyaibega village in the Bukoba rural district who was engaged in fishing on Goziba Island, ≈80 km east of Lake Victoria shore (Figure 1). The outbreak primarily affected Butahyaibega village in Kanyangereko ward and Bulinda village in Maruku ward, both located ≈20 km south of the town of Bukoba. That area is notable for its numerous mining sites and caves that are inhabited by colonies of the Egyptian rousette bat (*R. aegyptiacus*).

The probable index case for the 2025 MVD outbreak was in a resident of Katerela village in Ruziba ward of Biharamulo district, situated in proximity to the Burigi and Biharamulo game reserves and ≈170 km south of the 2023 outbreak location (Figure 1). The 2025 outbreak was confined to the 2 neighboring villages of Katerela and Ruziba; contacts and cases were reported primarily within Biharamulo and Muleba districts in the Kagera region. Of note, a total of 8 probable case-patients died before sampling could be conducted. Those case-patients were epidemiologically linked to 2 laboratory-confirmed cases, both of whom died.

### Sample Collection

During the MVD outbreaks, we collected blood samples in EDTA tubes for 84 suspected cases in 2023 and 81 suspected cases in 2025. We transported samples to a mobile laboratory stationed at Kabyaile Health Center in Missenyi district (Figure 1), which had been positioned in Kagera region as part of preparedness and response to the 2022–2023 Sudan Ebola virus outbreak in Uganda.

### Confirmation of MARV by Real-Time Reverse Transcription PCR

We tested blood samples for filoviruses Ebola virus (EBOV) and MARV using real-time reverse transcription PCR (RT-PCR). In brief, we extracted viral RNA from inactivated EDTA plasma collected using the QIAamp viral RNA mini kit (QIAGEN, https://www.qiagen.com), in accordance with the manufacturer’s instructions. The extracted RNA served as a template for the diagnosis of filoviruses using the RealStar filovirus screen RT-PCR kit 1.0 (Altona Diagnostics, https://altona-diagnostics.com) using a CFX96 real-time PCR detection system (Bio-Rad Laboratories, https://www.bio-rad.com), in accordance with manufacturer’s instructions. We conducted the initial real-time RT-PCR screening for both MARV and EBOV at the Kabyaile mobile laboratory before confirmation, cryopreservation, and next-generation sequencing at the national public health laboratory in Dar es Salaam.

### Sequencing Library Preparation and Next-Generation Sequencing of MARV

We prepared sequencing libraries using the Illumina viral surveillance panel (Illumina, https://www.illumina.com), following the Illumina RNA prep with enrichment protocol. We pooled, normalized, and quantified the resulting libraries using the Qubit DNA high sensitivity kit (Thermo Fisher Scientific, https://www.thermofisher.com). We performed paired-end sequencing using MiSeq (Illumina) with a MiSeq 600 cycle V3 kit and generated sequencing reads with a configuration of 2 × 150 bp, yielding an average of 11 million reads per sample.

### Bioinformatics Analysis

We subjected raw sequencing reads to quality control using FastQC version 0.11.9 (https://www.bioinformatics.babraham.ac.uk/projects/fastqc). We trimmed sequencing adapters and low-quality ends from reads using trim_galore version 0.6.4 (https://github.com/FelixKrueger/TrimGalore) powered by cutadapt version 4.5 (https://cutadapt.readthedocs.io/en/stable). We set the quality Phred score cutoff at 30 with a minimum read length of 75 bp. We aligned the quality-filtered sequencing reads to the isolate Marburg_virus/H.sapiens-tc/KEN/1980/Mt._Elgon-Musoke (GenBank accession no. NC_001608.3) as reference genome using Burrows-Wheeler Aligner version 0.7.17 with the maximum exact match (mem) algorithm (H. Li et al., unpub. data, https://doi.org/10.48550/arXiv.1303.3997). Subsequently, using the mapped sequencing reads, we performed de novo assembly using SPAdes version 3.13.1 (*17*) and Megahit version 1.2.9 (*18*), and evaluated the quality of the resulting assemblies using the Quality Assessment Tool (QUAST) program version 5.0.261 (*19*). We determined the MARV complete genome from each sample as the longest contig assembled.

For phylogenetic analysis, we aligned the MARV sequences generated in this study (deposited into GenBank under accession nos. PV700537–41) with those described previously available at GenBank by using MAFFT version 7.520 (https://mafft.cbrc.jp/alignment/software) to determine their evolutionary relationship (*20*). The alignment comprised 82 MARV complete genome sequences collected during 1967–2025 in Angola (n = 13), DRC (n = 25), Guinea (n = 2), Kenya (n = 6), the Netherlands (n = 1), Rwanda (n = 5), Sierra Leone (n = 3), Tanzania (n = 5), and Uganda (n = 22) (Appendix 1 Table). We used the model finder implemented in IQ-TREE version 1.6.12 (*21*) to determine the best-fitting model; we chose the general time reversible substitution model with estimated base frequencies, invariable sites, and gamma-distribution rate with 4 discrete categories in accordance with the Bayesian information criterion. We reconstructed a maximum-likelihood phylogenetic tree with IQ-TREE version 1.6.12 (*21*) and MEGA 12 using 1,000 bootstrap replications (*22*). We calculated the marginal likelihood estimation using Tracer version 1.7.2 (https://beast.community/tracer) to select the appropriate clock and demographic model. We used the uncorrelated relaxed clock with constant population model to generate the XML file in BEAUti version 1.10.4 (https://beast.community/beauti). We then analyzed the generated file using BEAST version 1.10.4 (https://github.com/beast-dev/beast-mcmc) for phylodynamic analysis, applying Monte Carlo Markov chain with a chain length of 20 × 10^6^, sampling after every 1,000 cycles. We explored the results using Tracer version 1.7.2, targeting an effective sample size of >200 for each parameter (*23*,*24*). We annotated the time-scaled phylogenetic tree generated by BEAST using TreeAnnotator version 1.10.4 (https://beast.community/treeannotator) with a maximum clade credibility tree after a burn-in of 20% of the sample; we used FigTree version 1.4.4 (https://tree.bio.ed.ac.uk/software/figtree) for visualization. We used the Nextstrain build for Marburg and Ravn viruses (https://github.com/pvanheus/nextstrain-marv-and-ravv) for ancestral state reconstruction and then the Auspice tool (https://docs.nextstrain.org/projects/auspice/en/stable/index.html) for visualization as described previously (*25*,*26*). We used the snp-sites tool (https://github.com/andrewjpage/snp_sites) to identify variable positions in the alignment after excluding unsequenced regions, and used snp-dists (https://github.com/tseemann/snp-dists) to count the single-nucleotide polymorphisms between and within outbreaks, as described previously (*27*).

## Results

### Comparative Genomics Analysis of MARV

We attempted virus enrichment sequencing using specimens from 10 Marburg virus–positive persons and generated whole genomes from 5 persons; 3 of those persons were from the 2023 outbreak (sample nos. S6, S7, and S8) and 2 from the 2025 outbreak (S001 and S002) (Table 1). The genomes of MARV strains reported in our study were 18,943–19,070 nt long (Table 1). With the exception of unsequenced regions between the MARV strains, there were no nucleotide sequence variations between strains from the same outbreak, whereas there were 39 single-nucleotide polymorphisms between the 2023 and 2025 MARV strains.

**Table 1:** Patient metadata and sequence metrics from study of 2023 and 2025 MARV outbreaks in Kagera region, northwestern Tanzania*

Characteristic	Patient 1	Patient 2	Patient 3	Patient 4	Patient 5
Sequence ID	MARV/H.Sapiens/TZ/Kagera/S6/2023	MARV/H.Sapiens/TZ/Kagera/S7/2023	MARV/H.Sapiens/TZ/Kagera/S8/2023	MARV/H.Sapiens/TZ/Kagera/001/2025	MARV/H.Sapiens/TZ/Kagera/002/2025
Patient age, y/sex	26/M	27/M	36/M	28/M	30/M
Location	Bulinda	Butahyaibega	Butahyaibega	Katerela	Ruziba
Ct value	24.41	18.73	19.74	25.28	16.34
Total sequencing reads	10,899,272	5,213,734	7,809,532	23,905,276	9,523,562
Mean base Phred quality score	35.4	35.2	35.4	37.4	37.3
No. MARV-specific sequencing reads	17,339	256,285	1,132,206	7,049,308	4,245,624
Mapped reads, %	0.16	4.91	14.46	29.49	44
Assembled MARV genome size, bp	18,943	19,054	19,048	19,070	19,030
GC content, %	38.28	38.18	38.07	38.10	38.01
Mean genome coverage depth	128.166	1,875.16	8,344.77	46,400	29,600
Genome coverage, %	99.12	99.70	99.67	99.89	99.57
GenBank accession no.	PV700539	PV700540	PV700541	PV700537	PV700538

*Ct, cycle threshold; GC, percentage of guanine (G) and cytosine (C) nucleotides; ID, identification; MARV, Marburg virus.

When we compared the 2023 and 2025 MARV genomes to a Marburg virus reference genome (GenBank accession no. NC_001608.3), we found 99.12%–99.89% genome coverage. BLASTn (https://blast.ncbi.nlm.nih.gov) revealed that Tanzania MARV strains were closely related to a MARV isolate collected from a bat in the Python Cave, southwestern Uganda, in 2009 (GenBank accession no. JX458855) and from a human during the September 2014 MVD outbreak in Kampala, Uganda (GenBank accession no. KP985768). When we compared those genomes to the MARV reference genome (GenBank accession no. NC_001608.3), mean coverage depth was 128.166–46,400, with variability along the genome (Table 1; Appendix 2 Figure 1). We observed >99.68% nucleotide identity between MARV strains responsible for the 2023 and 2025 outbreaks in Tanzania and those of 2024 outbreak in Rwanda (GenBank accession nos. PQ552725–42) (Table 2).

**Table 2:** Comparison of amino acid mutations in Marburg virus strains in study of Marburg virus disease outbreaks in 2023 and 2025, Tanzania*

Gene	2023 outbreak, Tanzania	2024 outbreak, Rwanda	2025 outbreak, Tanzania
VP35	I94V (A3224G)	I94V (A3224G)	I94V (A3224G)
VP40	V57I (G4736)	V57I (G4736), Q159R (5043G)	V57I (G4736)
GP	P234S (C6640T), Y279D (6775G), H349N (C6985), S389G (A7105G)	P234S (C6640T), Y279D (6775G)	P234S (C6640T), Y279D (6775G), E241G (A6662G), H349N (C6985), S389G (A7105G)
L	I243L (A12207C), D699N (G13575A), V1673A (T16494C), M1734V (16680G), R1756S (A16748C),	R1756S (A16748C)	K174R (A12001G), V1673A (T16494C), R1756S (A16748C), S1820L (C16939T, T16940A), R1855K (G17044A)

*Study strains were compared with a reference isolate, GenBank accession number NC_001608.3. GP, glycoprotein; L, polymerase; VP, viral protein.

### Phylogenetic Analysis

**Figure 2:**
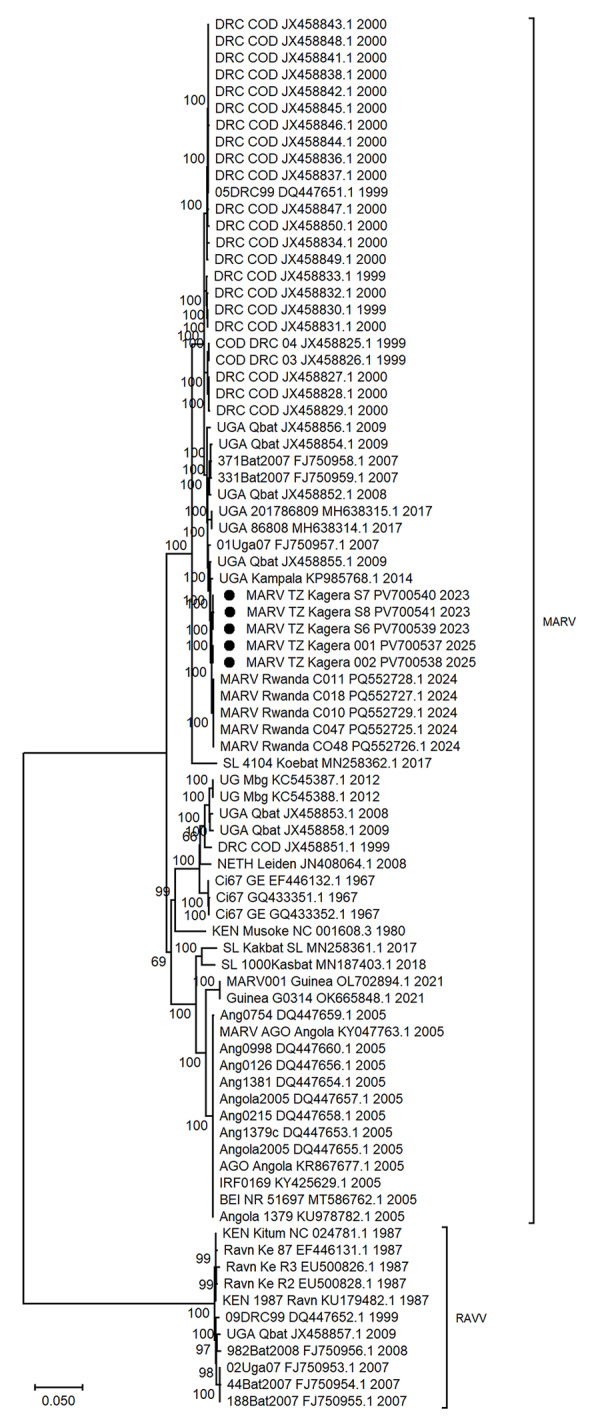
Maximum-likelihood phylogenetic tree from study of 2023 and 2025 MARV outbreaks in Kagera region, northwest Tanzania. Tree was reconstructed using 82 MARV complete genome sequences, including sequences responsible for the 2023 and 2025 outbreak in Tanzania described in this study (black dots) and previously reported sequences acquired from GenBank (accession numbers provided). Node values show the percentage of bootstrap support. Scale bar indicates nucleotide substitution per site. Ang, Angola; DRC, Democratic Republic of the Congo; KEN, Kenya; MARV, Marburg virus; NETH, the Netherlands; SL, Sierra Leone; UG or UGA, Uganda.

A comparison of Tanzania MARV sequences with all available full-length MARV sequences from previous outbreaks across Africa demonstrated that the 2023 and 2025 sequences were closely related and clustered with MARV sequences from outbreaks in Rwanda in 2024 (bootstrap support 100%) and Uganda in 2014 (bootstrap support 100%) (Figure 2). The sequences from both MVD outbreaks in Tanzania showed >99.71% nucleotide identity, suggesting a possible local single spillover event in each of the Bukoba Rural and Biharamulo districts, followed by limited human-to-human virus transmission. The clock likeness analysis using TempEst version 1.5.3 (https://tree.bio.ed.ac.uk/software/tempest) revealed the existence of a positive temporal signal among representative full-length MARV sequences with a correlation coefficient of 0.7184 and a coefficient of determination (R^2^) of 0.5161. The time to most recent common ancestor for the analyzed dataset was 1767 (95% highest posterior density interval range 1591–1966) and the evolution rate was 2.2233 × 10^−4^ (95% CI 1.8419 × 10^−4^ to 2.634 × 10^−4^) substitutions/site/year. Bayesian evolutionary tree reconstruction analysis revealed that the Tanzania MARV strains clustered with the 2024 Rwanda MARV strains. In addition, the MARV strains from Tanzania were closely related to those collected in Uganda from bats in 2009 and humans in 2014.

## Discussion

Marburg virus disease is a VHF with dramatic clinical manifestation and a high CFR that poses a substantial global health threat and negative economic consequences (*28*). The 3 MVD outbreaks reported in 2023, 2024 and 2025 in Tanzania and Rwanda occurred in a radius of ≈150 km, underscoring the common ecologic zone (Figure 1, panel A). We did not observe nucleotide substitutions in MARV from the same MVD outbreaks in Tanzania. Although the specific evolutionary origins of MARV in the Great Lakes ecosystem remains unresolved, we noted that the probable index cases in the Tanzania outbreaks in 2023 and 2025 were residents within the Kagera region with no foreign travel history. The Kagera region, where the Tanzania MVD outbreaks occurred, borders Uganda to the north, Rwanda to the west, and Burundi to the southwest; most of the area forms the Kagera River basin ecosystem, which is ecologically linked to the Albertine Rift montane forest ecoregion and the Greater Virunga Landscape. The Kagera River basin represents an interface of the highest biodiversity and diverse agro-ecosystems of Africa covering 5 countries; Burundi, DRC, Rwanda, Uganda, and Tanzania. High population mobility and diverse rich flora and fauna within the Kagera River basin ecosystem increase the risk for pathogen spillover and cross-border transmission of infectious diseases, including MVD. In East Africa, the distribution of Egyptian rousette bats encompasses Uganda, Rwanda, Burundi, and parts of the DRC, Kenya, and Tanzania. Although no study has reported MARV in bats in Tanzania, the virus has been isolated from bats in Uganda, DRC and Kenya (*9*,*29*,*30*). The area in Tanzania in which the 2023 and 2025 MVD outbreaks occurred is notable for its numerous mining sites and caves that are inhabited by colonies of Egyptian rousette bats, the reservoir host for MARV (*31*). The nucleotide sequences from both outbreaks were highly similar, suggesting a possible local single spillover event followed by limited human-to-human virus transmission.

The village where MVD was reported for the first time in Tanzania is the location of the Kanyangereko Cave, one of the largest Egyptian rousette bat roosting sites in the Kagera region, suggesting a likely source of the virus (*31*). High-risk human behavior and practices, including collection of bat-derived manure (guano) from caves for crop fertilization and direct contact with fruits partially eaten by Egyptian rousette bats, have been reported in the region, increasing the MARV spillover risk to humans (*9**,**31*). The possibility of virus dispersion from Kagera to other location in Tanzania or neighboring countries through movement of possibly infected bats cannot be excluded; average flight distances of 60.18 km for Egyptian rousette bats and interactions with other animal species and humans have been documented (*9*). 

Reported MVD outbreaks in Africa, including within eastern Africa, highlight the growing MVD public threat (*32*). It is difficult to determine whether the number of MARV outbreaks has increased in recent years or if more cases have been identified through improved diagnostics capacity and public health surveillance systems. Investigation of MARV in Egyptian rousette bats in Tanzania is crucial to understand the extent of the risk posed to the human population in the Kagera region and beyond. The fact that the exact origin of the MVD outbreaks in Tanzania is not identified poses a continued threat to the population in the Kagera River basin. We recommend anthropologic and ecologic studies to identify the virus reservoirs and factors that enhance MVD transmission dynamics in this ecosystem.

Our findings indicated that genetically closely related MARV strains were responsible for the 2023 and 2025 MVD outbreak in Kagera region of Tanzania, which supports the hypothesis of a local zoonotic spillover event followed by limited human-to-human virus transmission. More MARV complete genomes from humans and bats and other reservoirs from eastern Africa countries are needed to accurately understand the evolution and spread pattern of MARV, which can help public health authorities to design effective prevention and control strategies. Furthermore, obtaining complete genome sequences during outbreaks are crucial for effective monitoring, management of new waves of infection, and improved understanding of the transmission dynamics.

Appendix 1 Additional data from study of Marburg virus from outbreaks in 2023 and 2025 in Kagera, Tanzania.

Appendix 2 Additional information about study of Marburg virus from outbreaks in 2023 and 2025 in Kagera, Tanzania.
