# Supplementary material for: Genomic Insights into Marburg Virus Strains from 2023 and 2025 Outbreaks in Kagera, Tanzania
Source: Emerg Infect Dis. 2026 Jan;32(1):55–62. doi: 10.3201/eid3201.251314 (PMC12869988; doi:10.3201/eid3201.251314)
Supplement: Appendix 2 — Additional information about study of Marburg virus from outbreaks in 2023 and 2025 in Kagera, Tanzania. [file 25-1314-Techapp-s2.pdf]

Article DOI: https://doi.org/10.3201/eid3201.251314

EID cannot ensure accessibility for supplementary materials supplied by authors. Readers who have difficulty accessing supplementary content should contact the authors for assistance.

Genomic Insights into Marburg Virus Strains from 2023 and 2025 Outbreaks in Kagera, Tanzania

Appendix 2

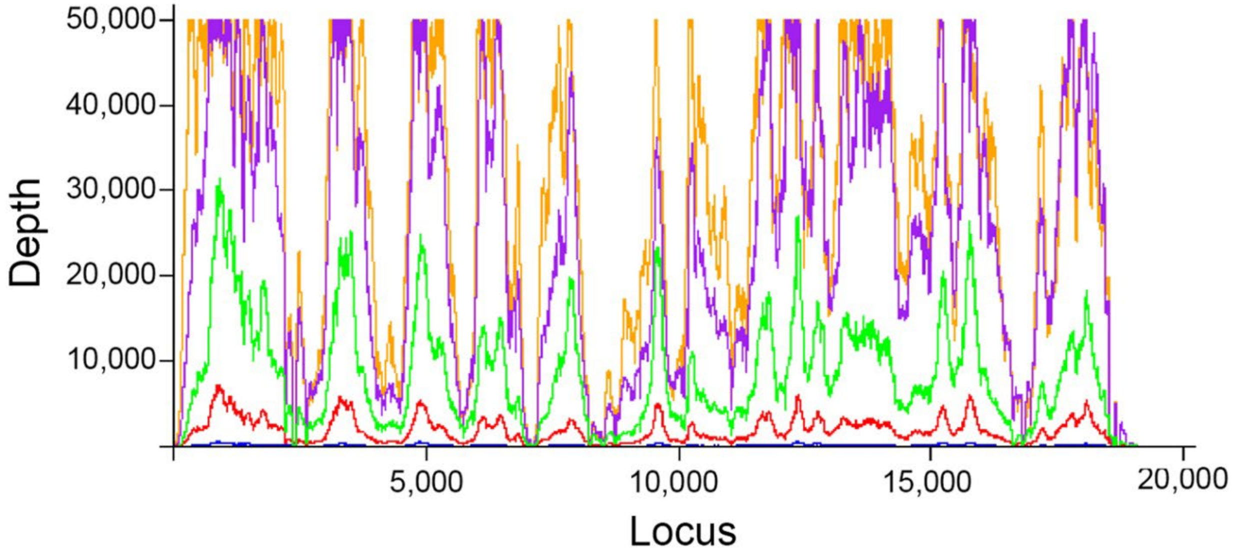

Appendix 2 Figure. Comparative genomic analysis of the Marburg viruses from Tanzania. The figure depicts the presentation of the coverage depth of the MARV/H. Sapiens/TZ/Kagera/S6/2023 (blue), S7/2023 (red), S8/2023 (green), 001/2025 (orange) and 002/2025 (purple) described in this study after mapping reads to a MARV reference genome (NC001608.3). The coverage was determined using SAM tools version 1.10 and visualized with the lattice package in R Studio. The x-axis indicates the locus on the reference genome in base pairs. The depths of sequences 001/2025 and 002/2025 were truncated as they exceeded sequencing depth of 50,000 for some loci.
